# Modified ventriculoperitoneal shunt applied to temporary external ventricular drainage

**DOI:** 10.1038/s41598-024-66917-x

**Published:** 2024-07-11

**Authors:** Zhixiong Lin, Jintao Chen, Weili Lin, Bei Liu, Chaoqun Weng, Yongzhao Yang, Congai Liu, Rongbiao Zhang

**Affiliations:** 1 Department of Neurosurgery, Sanbo Brain Hospital, Capital Medical University, Xiangshanyikesong 50#, HaiDian District, Beijing, China; 2 Department of Neurosurgery, Fujian Sanbo Funeng Brain Hospital, Fuzhou, Fujian China

**Keywords:** Hydrocephalus, External ventricular drainage, Ommaya reservoir, Modified ventriculoperitoneal shunt, Neurology, Hydrocephalus

## Abstract

External ventricular drainage (EVD) is a common procedure in neurosurgical practice. Presently, the three methods used most often include direct EVD (dEVD), long-tunneled external ventricular drains (LTEVDs), and EVD via the Ommaya reservoir (EVDvOR). But they possess drawbacks such as limited duration of retention, vulnerability to iatrogenic secondary infections, and challenges in regulating drainage flow. This study aimed to explore the use of a modified ventriculoperitoneal shunt (mVPS)—the abdominal end of the VPS device was placed externally—as a means of temporary EVD to address the aforementioned limitations. This retrospective cohort study, included 120 cases requiring EVD. dEVD was performed for 31 cases, EVDvOR for 54 cases (including 8 cases with previously performed dEVD), and mVPS for 35 cases (including 6 cases with previously performed EVDvOR). The one-time success rate (no need for further other EVD intervention) for dEVD, EVDvOR, and mVPS were 70.97%, 88.89%, and 91.42%, dEVD vs EVDvOR (*P* < 0.05), dEVD vs mVPS (*P* < 0.05), EVDvOR vs mVPS (*P* > 0.05). Puncture needle displacement or detachment was observed in nearly all cases of EVDvOR, while no such complications have been observed with mVPS. Apart from this complication, the incidence of postoperative complications was 35.48%, 14.81%, and 8.5%, dEVD vs EVDvOR (*P* < 0.05), dEVD vs mVPS (*P* < 0.05), EVDvOR vs mVPS (*P* > 0.05). Mean postoperative retention for EVD was 14.68 ± 9.50 days, 25.96 ± 15.14 days, and 82.43 ± 64.45 days, respectively (*P* < 0.001). In conclusion, mVPS significantly extends the duration of EVD, which is particularly beneficial for patients requiring long-term EVD.

## Introduction

External ventricular drainage (EVD) is a common technique in neurosurgery. It is used most frequently in patients with progressive hydrocephalus who are unable to experience self-improvement or undergo curative surgery (including ventriculostomy or common shunt surgery) for hydrocephalus due to pathological factors associated with abnormal cerebrospinal fluid (CSF) properties over an extended period of time. Such cases represent approximately 5% of all cases of hydrocephalus^1,2^. In addition, EVD is frequently observed in cases of hydrocephalus induced by diverse brain tumors, serving as a provisional intervention prior to the achievement of direct tumor resection^3,4^.

At present, three methods are commonly used for EVD, including direct EVD (dEVD) (conventional 5 cm tunneling), long-tunneled external ventricular drains (LTEVDs) (conventional > 5 cm tunneling), and EVD via Ommaya reservoir (EVDvOR)^3–5^, with a limited indwelling time of 7–10 days^6–9^, which poses challenges in effectively regulating the drainage volume^10^. Although EVDvOR can be placed for a long time by replacing the scalp puncture, caring for patients in clinical practice presents challenges, as does the fixation of the Ommaya reservoir following puncture, the vulnerability of the needle to displacement and detachment, as well as the increased patient discomfort, clinical cost and risk of infection associated with repeated punctures; sometimes requiring secondary operations to replace the drainage site. While EVDvOR and LTEVDs can prolong drainage time and significantly reduce the risk of infection, challenges exist in terms of managing drainage flow, patient activities, and clinical management, thereby hindering the fulfillment of clinical requirements. Consequently, there is a pressing demand for a novel approach that can effectively control hydrocephalus, while being easy to use, resistant to infection, and readily accepted by family members, particularly in patients with intracranial infection combind hydrocephalus and/or high protein content in CSF^11^, who can no longer undergo conventional hydrocephalus shunt for a short period of time, and need long-term EVD^12–14^.

In recent years, our research has focused on investigating an mVPS—The abdominal end of the VPS device was placed externally—building upon the use of dEVD and EVDvOR. This surgical approach has been shown to effectively address the aforementioned limitations associated with the procedures. In this study, we retrospectively analyzed the success rates, complication rates, average indwelling times, and treatment costs associated with three EVD surgical procedures. The objective of this study was to comprehensively analyze and compare various temporary EVD to determine the most efficacious surgery for patients.

## Methods

### Study design

This study is a retrospective, observational, single-center cohort study. The study was reviewed and approved by the human subjects’ institutional review boards (FJSBNK-YJ-2024-001-02). All included patients provided signed informed consent to participate in the study. The patient consented to the publication of his/her image. The study were performed in accordance with relevant guidelines and regulations.

### Selection of study population

This study collected data of 120 consecutive cases who underwent surgical intervention in our institution from August 2017 to December 2023 for various etiologies causing hydrocephalus which required temporary external CSF drainage surgery.

This dataset summarizes the patients' baseline demographic and clinical characteristics, including age, sex, symptoms, causes of hydrocephalus, one-time success rate of operation, tube placement time, cost of surgery, and whether secondary infections occurred, among other complications.

Inclusion criteria were as follows: (1) Patients with intracranial infection occurring after VPS or other external drainage for hydrocephalus; (2) Patients with intracranial infection accompanied by acute hydrocephalus subsequent to other intracranial surgical procedures; (3) Patients for whom CT/MRI suggested acute obstructive hydrocephalus with ventriculomegaly resulting from cerebral hemorrhage; (4) Patients with hydrocephalus caused by a tumor or other etiology who need drainage treatment.

Exclusion criteria were: (1) Patients unable to withstand surgery due to poor nutritional status; (2) Patients with severe coagulopathy.

### Surgical methods

**Figure 1:**
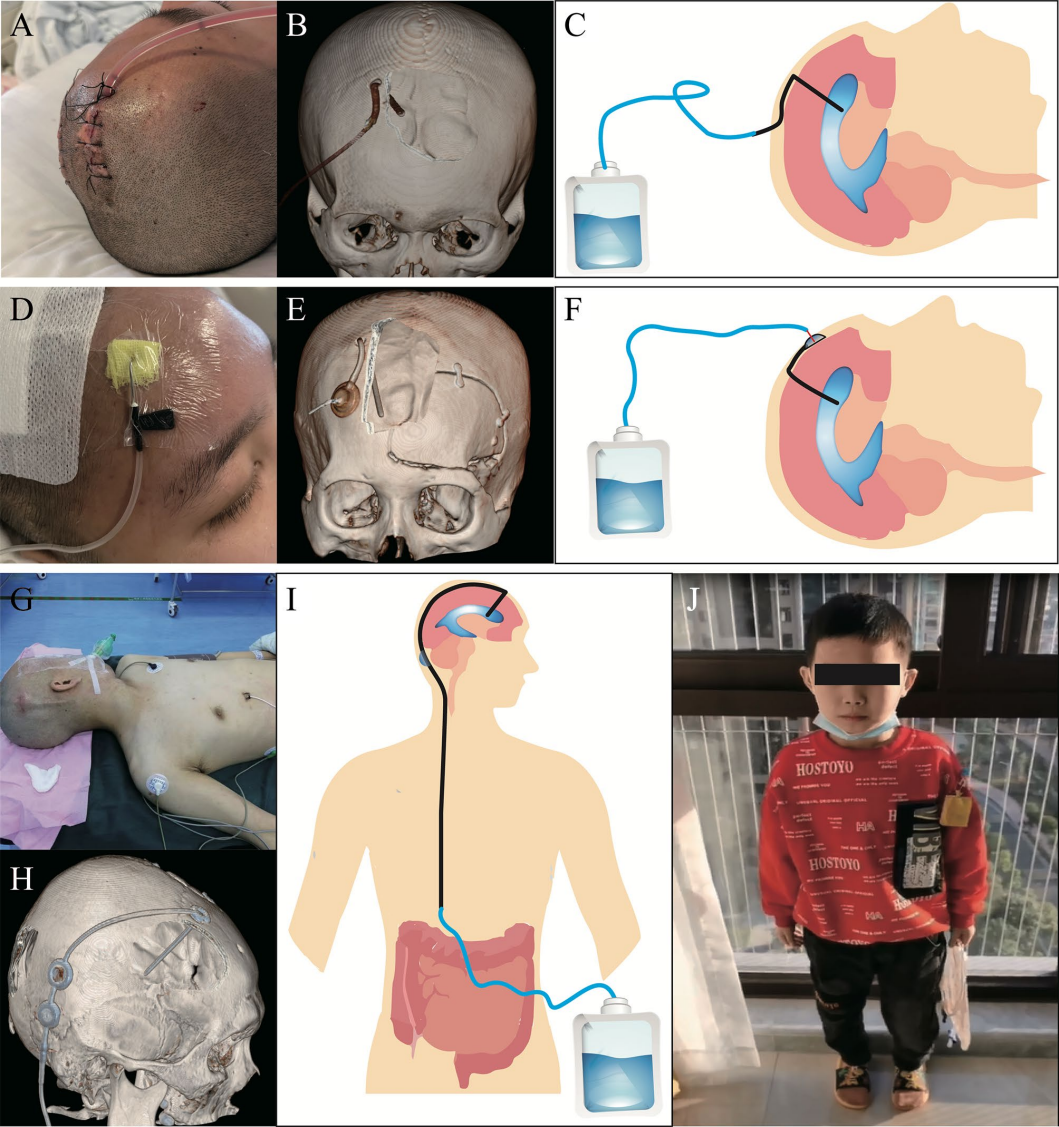
Schematic diagram of three external ventricular drainage procedures. The actual picture, drainage tube reconstruction picture and simulation picture of direct external ventricular drainage are A/B/C, respectively; The actual picture, drainage tube reconstruction picture and simulation picture of external ventricular drainage with Ommaya reservoir are D/E/F, respectively; The actual picture, drainage tube reconstruction picture and simulation picture of ventriculoperitoneal shunt external peritoneal drainage are G/H/I/J, respectively.

All patients underwent EVD under general anesthesia. After intubation under general anesthesia, patients were in a supine position with heads tilted to the right (left) side. (1) dEVD was performed with an ordinary 12-measure drainage tube, and the sterile extracorporeal drainage bag was attached after routine surgery (the sterile drainage bag was replaced frequently); (2) The other two techniques used Aesculap (Aesculap Inc., Center Valley, PA, USA) or Medtronic (Medtronic Instruments Inc., Minneapolis, MN, USA) CSF shunt tubes and kit, as follows: A) EVDvOR: After successful ventricle end puncture of shunt tube, the ommaya reservoir was connected, and then the ommaya reservoir was buried under the front forehead. At the same time, percutaneous puncture of the ommaya reservoir was performed using scalp needle and a sterile drainage bag was connected (scalp needle and sterile drainage bag were replaced routinely every 3 days). B) mVPS: the shunt tube was connected to the fluid storage sac and the gravity valve of the medium pressure tube after successful ventricular end puncture. Peritoneal end: the peritoneal catheter was tunneled subcutaneously to the lower chest/upper abdomen, where it was then externalized through a small skin incision and connected to an external sterile drainage bag. The control of drainage flow was achieved through the utilization of the gravity valve located within the shunt tube, which was positioned at the auricle level in relation to the ventricle. The average rate of CSF flow was regulated to approximately 10 mL/h, while it is advised that the upper limit for drainage flow should not exceed 300 mL per day. (Fig. 1) To control the flow, it is not recommended an mVPS without a valve.

The selection of the surgical procedure was based on the preferences expressed by the diagnosis, etiologies, and condition judgment predicted the need for catheterization for more than 2 weeks. In general, we would recommend mVPS first, followed by EVDvOR or LTEVDs, and ultimately dEVD.

### Curative effect evaluation standard

The complications rates, length of drainage tubes, and one-time success rates (no need for further other EVD intervention) of the treatment processes of dEVD, EVDvOR, and mVPS. Additionally, the efficacy evaluation indicators including EVD, nursing drainage tube, the cost of late complications treatment, Evans index, Glasgow Coma Scale (GCS) scores, and Glasgow Outcome Score (GOS) grades before and after treatment (at discharge) were evaluated. Upon discharge, patients’ outcomes were evaluated using the GOS classification, with a score of 4–5 indicating a favorable recovery and a score of 3 or less indicating a poor recovery outcome.

### Patient follow-up

All patients were followed through outpatient examinations, telephone interviews and WeChat. In addition, follow-up included nervous system physical examination, head CT or MRI, with follow-up time ranging from 6 months to 5 years.

### Statistical Analysis

All statistical analyses were conducted using SPSS software (IBM SPSS Statistics Version 24.0, IBM Corp., Armonk, NY, USA), and the measurement data consistent with the normal distribution were expressed as (mean ± standard deviation). The measurement data for non-normal distributions were expressed as medians, and non-parametric tests were applied. Enumeration data were assessed by the χ^2^ test. Jonckheere–Terpstra test was used to explore the mean drainage time. Categorical variables, such as sex, clinical symptoms, and etiology, are presented using frequency and percentage descriptions. *P* < 0.05 indicated statistically significant difference.

## Results

### Patient Characteristics

**Figure 2:**
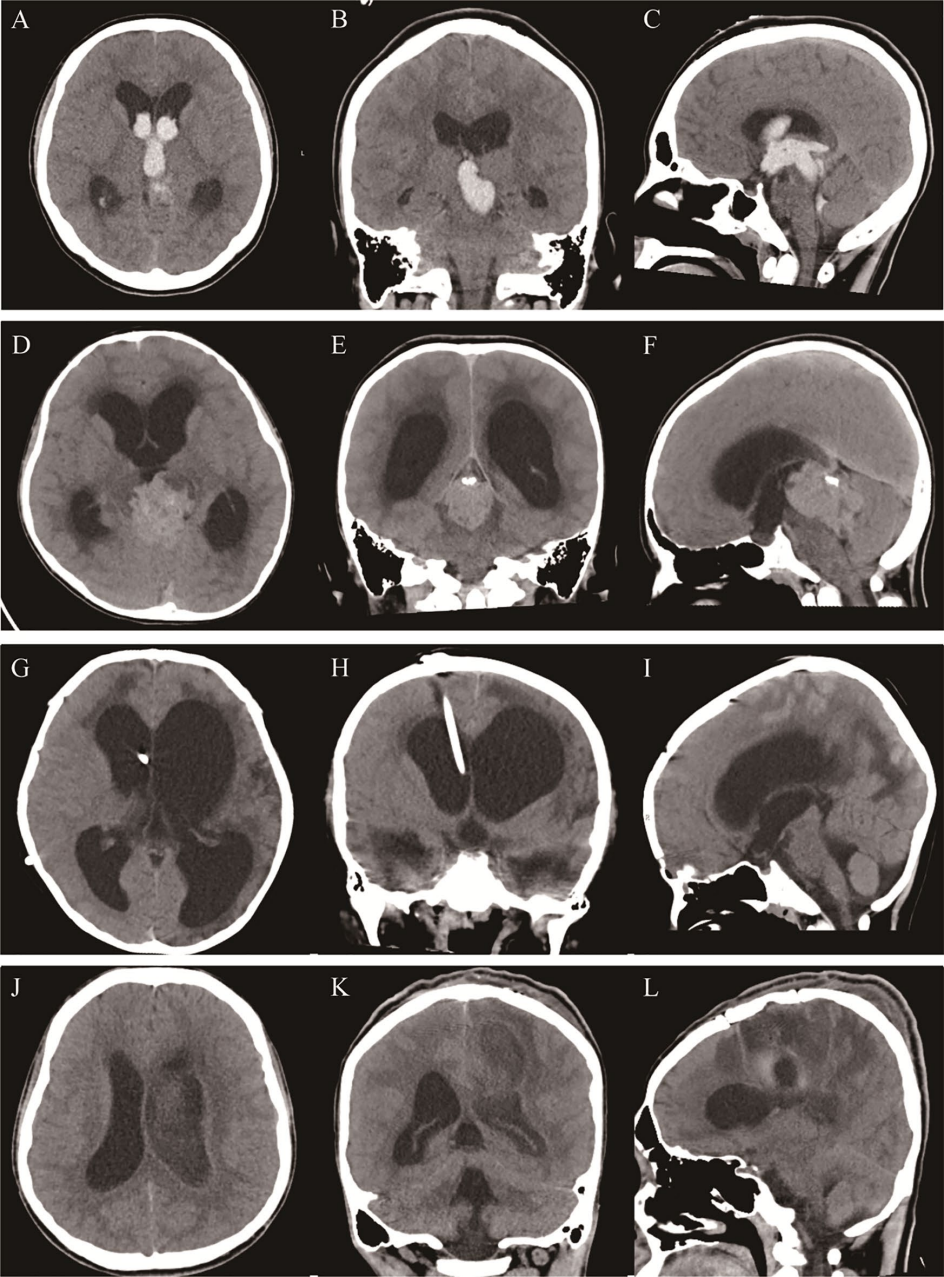
CT imaging features of various acquired hydrocephalus. Imaging of secondary hydrocephalus due to intraventricular hemorrhage: Axial (**A**), coronal (**B**), and sagittal (**C**), Imaging of obstructive hydrocephalus caused by brain tumor: Axial (**D**), coronal (**E**), and sagittal (**F**), Imaging of secondary intracranial infection after ventriculoperitoneal shunt: Axial (**G**), coronal (**H**), and sagittal (**I**), Imaging of brain abscess with intracranial infection: Axial (**J**), coronal (**K**), and sagittal (**L**).

The study cohort consisted of 120 cases (Table 1) and included 79 males (65.8%, range 1.42–78 years) and 41 females (34.2%, range 1.67–87 years). Among these, 22 cases had acute obstructive hydrocephalus due to cerebral hemorrhage breaking into ventricles; 34 cases had obstructive hydrocephalus due to tumor compression in posterior cranial fossa, ventricles or pineal body region requiring continuous EVD of CSF; 64 cases had intracranial infection complicated with hydrocephalus due to various etiologies, including 18 cases of intracranial infection after VPS, 27 cases of intracranial infection complicated with hydrocephalus after cerebral hemorrhage, and 3 cases of primary intracranial abscess, tuberculous encephalitis complicated with hydrocephalus. Encephalitis complicated with hydrocephalus and cryptococcus complicated with hydrocephalus were 1 case each. Imaging features of various acquired hydrocephalus are shown in Fig. 2.

**Table 1:** Patient characteristics.

Characteristic	Value
Sex	
Male	79/120(65.8%)
Female	41/120(34.2%)
Overall age(range) (years)	34.49 (1.42–87)
Within operation(cases)	120/120(100.00%)
Clinical symptoms	
Symptoms of cranial hypertension	120/120(100%)
Evans Index	0.42 ± 0.07
Posthemorrhagic hydrocephalus	22/120(18.33%)
Tumor	34/120(28.33%)
Secondary intracranial infection with hydrocephalus	64/120(53.33%)

### One-time success rates, complications and indwelling time

**Figure 3:**
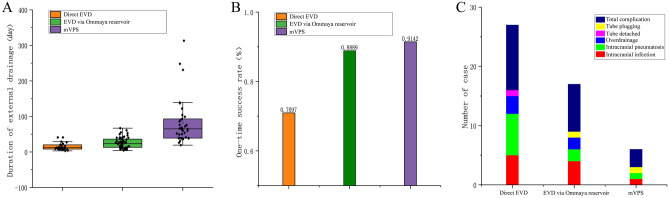
Comparison of the success rate of one-time target control (**A**), complications (**B**) and indwelling time (**C**) between the three kinds of external ventricular drainage.

Comparison of the one-time success rates (no need for further other EVD intervention) for the three operations—dEVD, EVDvOR, and mVPS—showed success rates of one-time procedures for hydrocephalus and related targets, respectively, as: 70.97% (22, 31), 88.89% (48, 54), and 91.42% (32, 35), (dEVD vs EVDvOR: χ = 4.352, *P* = 0.037; dEVD vs mVPS: χ = 4.626, *P* = 0.031, EVDvOR vs mVPS: χ = 3.54, *P* = 0.698, Fig. 3).

In the cohort of dEVD failed among 9 cases, complications were observed, including intracranial infections in 5 cases, tube detachment in 1 case, and persistent obstructive hydrocephalus despite drainage exceeding two weeks in 3 cases. Among the 6 cases in which EVDvOR failed, 4 cases of intracranial infections, 1 case of tube blockage, and 1 case of skin damage resulting from repeated punctures to the OR. In the cohort of mVPS failure among 3 cases, 2 cases experienced recurrent intracranial inflammation. This included 1 case of re-emergent Staphylococcus epidermidis infection and another case associated with poorly controlled Klebsiella pneumoniae infection, which was linked to recurrent pneumonia. Additionally, 1 occurrence of tube blockage was observed.

In this dataset concerning OR, the analysis excluded complications related to needle displacement and detachment during the puncture procedure, instead concentrating on complications stemming from detachment of the drainage tube from the ventricular end, due to the occurrence of needle displacement and dislodgement following OR puncture in all cases. The incidence of postoperative complications of dEVD, EVDvOR, and mVPS were 35.48% (11/31), 14.81% (8/54), and 8.5% (3/35) (dEVD vs EVDvOR: χ = 4.848, *P* = 0.028; dEVD vs mVPS: χ = 7.124, *P* = 0.008, EVDvOR vs mVPS: χ = 4.33, *P* = 0.382). There was no significant difference regarding intracranial infections between EVDvOR and mVPS (χ = 0.193, *P* > 0.05). Complications of dEVD included intracranial infection in 5 cases, intracranial pneumatosis in 7 cases (including 3 cases with intracranial infection), overdrainage in 3 cases (including 2 cases with intracranial pneumatosis, overdrainage refers to slit ventricle and subdural effusion), and tube detachment in 1 patient. In EVDvOR, there were 4 cases of intracranial infection, 2 cases of intracranial pneumatosis, 2 cases of overdrainage (only) and 1 case of tube blockage. In mVPS, there was 1 case of intracranial infection, 1 case of pneumatosis and 1 case of tube plugging in the external placement of the ventriculoperitoneal end (Table 2).

**Table 2:** Comparison of complications associated with the three treatment methods.

Complications	Direct EVD (n = 31)	EVD via Ommaya reservoir (n = 54)		mVPS (n = 35)
Intracranial infection	2		3	1
Intracranial pneumatosis	2		1	1
Intracranial pneumatosis with infection	3		1	0
Intracranial pneumatosis with overdrainage	2		0	0
Overdrainage	1		2	0
Tube detached	1		0	0
Tube plugging	0		1	1
Total	11		8	3

The mean drainage time of direct ventricular drainage surgery was 14.68 ± 9.50 days, ranging from 3 to 41 days; the mean drainage time of EVDvOR was 25.96 ± 15.14 days, ranging from 4 to 67 days; the mean drainage time of mVPS was 82.43 ± 64.45 days, ranging from 19 to 313 days the CSF was purified to a normal state, patients with an average drainage duration of over 100 days are all patients with intracranial infections (*P* < 0.001).

Furthermore, mVPS can prevent overdrainage in patients during activity by regulating the flow through the diversion tube valve, contrasting with the limitations imposed by dEVD and EVDvOR which not only impede patient activity but also contribute to increased drainage in cases of improper patient positioning.

### Cost of EVD operation

Because most patients were complicated with comorbid diseases, the present study only counted the nursing and operation costs for hydrocephalus, in which the nursing and operation cost of dEVD was the lowest, while the overall operation cost of mVPS was the highest, and the comparison of the total costs between the three operations was statistically significant. However, if the first operation is unsuccessful, one of the above three operations needs to be performed again, so the overall cost will be doubled or more. In this study, 8 cases failed to perform dEVD for the first time, (Five cases of secondary intracranial infection resulting from dEVD, 2 intracerebral hemorrhage with obstructive hydrocephalus that didn't be alleviated in the short term, and 1extubation), and these patients were underwent EVDvOR again. 6 other cases failed to perform EVDvOR and underwent mVPS again (Table 3). The enhancement of the one-time success rate also resulted in a decrease in hospital stays attributable to recurrent surgical interventions, which averaged approximately 30 days.

**Table 3:** Comparison of the treatment costs between the three groups (x ± s).

	N	Cost of operation (ten thousand, RMB)	*P*	Cost of nursing EVD (ten thousand, RMB)	*P*
Direct EVD	31	0.43 ± 0.06	P* < 0.01	0.11 ± 0.05	*P*^*1*^ < 0.05 *P*^*2*^ > 0.05
EVD via OR	54 (8)	0.65 ± 0.11		0.23 ± 0.10	
mVPS	35 (6)	1.24 ± 0.16		0.21 ± 0.09	
At least two or more operations	8 + 6 (14)	1.87 ± 0.33^#^	*P*^&^ < 0.05	0.36 ± 0.13	*P*^*3*^ < 0.05

^#^A total of 14 cases underwent two or more operations in the same case, of which 8 cases underwent EVD via Ommaya reservoir again after failure of the first Direct EVD, and 6 cases underwent mVPS again after EVD via Ommaya reservoir.

*The comparison of treatment cost between mVPS and Direct EVD, mVPS and EVD via Ommaya reservoir was statistically significant (*P* < 0.01). & The cost of comparing mVPS and multiple operations was statistically significant (*p* < 0.05). The statistical significance of the external drainage nursing costs is assessed between Direct EVD and EVD via OR, Direct EVD and mVPS, and mVPS and multiple operations, denoted as *P*^1^, *P*^2^, and *P*^3^, respectively (*P* < 0.05).

Furthermore, some patients experience relief from hydrocephalus after a period of time, eliminating the need for further shunt surgery. The cost associated with the removal of drainage devices was found to be consistent across patients undergoing dEVD, EVD, and mVPS. The cost associated with decreases in hospital stays was not evaluated at this time.

### Patient outcomes

After treatment, the Evans index and GCS scores of patients were significantly improved. According to GOS classification at discharge, good recovery (4–5 points) of dEVD, EVDvOR and mVPS were 64.52% (20, 31), 72.22% (39, 54) and 62.86% (22, 35), respectively (*P* > 0.05), and poor recovery (3 points) of the three surgeries were 35.48% (11, 31), 27.78% (15, 54) and 37.14% (13, 35), respectively (*P* > 0.05). No statistically significant differences were found among the three surgeries (GOS score was 3 points at admission, 13 cases had intracranial infection complicated with hydrocephalus after cerebral hemorrhage surgery, and 9 cases had hydrocephalus after VPS). There was no difference in infection after permanent shunt following external drainage between groups.

120 cases underwent external drainage, 44 cases had no ventricular dilatation and no hydrocephalus on CT scan, and the EVD tubes were removed. The other 76 cases could not be relieved because of hydrocephalus, when CSF characteristics were consistent with permanent VPS.

Among the 35 cases with mVPS, 28 cases underwent permanent VPS. In most cases, only the peritoneal catheter is replaced in patients with permanent VPS, unless the patient specifically asks for a brand new one; 6 cases (5 children) didn’t find hydrocephalus after mVPS. So, the visible portion of the abdominal cavity can be ligated and subsequently hidden under the skin in a similar Ommaya reservoir treatment, thus avoiding the need for subsequent surgical removal. One case gave up treatment because of brain tumor metastasis and aggravation.

## Discussion

This study examined the efficacy of dEVD, EVDvOR, and mVPS in treating temporary external CSF drainage surgery resulting from diverse etiologies. The comparison of one-time success rates (no need for further other EVD intervention), complication rates, and indwelling time revealed that mVPS exhibited a high one-time success rate, fewer subsequent complications, and a longer indwelling time; Although mVPS has only a small innovation, the abdominal end of the shunt is placed outside. But, the mVPS is secure, and dependable, offering numerous advantages to patients.

Early CSF drainage relieves acute intracranial hypertension and hydrocephalus, avoids the occurrence of cerebral hernia, restores the shape of ventricle and brain tissue, and restores neurological damage to the greatest extent possible. Temporary EVDs, which include dEVD, LTEVDs and EVDvOR^3–5^, are also available in cases of cerebral hemorrhage or infection and external lumbar cisternal drainage^15^. Although the operation is convenient, it is associated with postoperative care difficulties, including short indwelling time, easily pulled out, causing iatrogenic secondary infection, restricting patient activity, and difficulty of controlling the flow of drainage.

The mVPS can greatly prolong the time of external drainage. The average duration of drainage was 83.74 days and the longest 313 days. Two patients are even able to carry the drainage tube to undergo hospital outpatient disinfection every week after discharge, including regular replacement of the drainage bag, reducing the economic burden, allowing patient activity and reducing hospitalization days. After the routine and biochemical results for CSF become normal, the next phase of the treatment plan is hospitalization. In the present study, among 35 cases, only 1 (2.86%) had secondary infection. Not only was the indwelling time much longer than that of the traditional external drainage, but it was also much lower than that reported previously 5.08–13.3%^13,16^. Therefore, this temporary drainage system allows long-term drainage of the infected CSF until the CSF cell count becomes normal and the protein levels return to normal. High CSF protein levels can lead to shunt failure. In a study involving 214 children and 376 adults who underwent VPS for tuberculous meningitis, the incidences of VPS obstruction were 27.5% and 25.5%^11^, respectively. However, the failure rate due to prolonged catheter placement in this cohort was 7.14% (2/28).

Four factors observed in this study help explain why this surgical procedure can place EVDs for a long time without infection, including: (1) Shunt placement by subcutaneous sheath extends the export distance of the drainage tube from the scalp to the cranial cavity, and reduces the chance of the cranial cavity being in direct contact with the outside world, coupled with the distance extended after strengthening the scalp itself, plays a role in immune resistance to infection, according to a new study^4,13^, tunneling using a ventricular drainage tube subcutaneous sheath longer than 5 cm can significantly reduce the infection rate. Consequently, the longer the tunneling distance, the lower the infection rate^17–21^. (2) External drainage reduces the chance of CSF leakage from the incision and avoids causing retrograde intracranial infection^22,23^. (3) The shunt was placed subcutaneously and fixed on the scalp, with a reservoir and shunt pump attached to the head to prevent accidental detachment of the drainage tube, thus prevented the failure of external drainage and intracranial infection caused by repeated reinsertion of the drainage tube. (4) The shunt pump's unidirectional valve with anti-reflux properties effectively prevents retrograde intracranial infection resulting from cerebrospinal fluid reflux.

Furthermore, mVPS can prevent overdrainage in patients during activity as the presence of the valve, contrasting with the limitations imposed by dEVD EVDvOR, and LTEVDs which not only impede patient activity but also contribute to increased drainage in cases of improper patient positioning.

Previous studies have also suggested that long time EVD was safe and effective in the treatment of patients with pyogenic ventriculitis and intracranial tuberculosis infection^14,17^. This method uses a special shunt device to draw the ventricular drainage tube from the chest or upper abdomen through the subcutaneous tunnel. It is suitable for patients with hydrocephalus caused by various etiologies who do not want or are not suitable for shunt in the short term. The indwelling time for external abdominal cavity drainage tubes is long, CSF long-range drainage results in reduced CSF protein fiber, reduced levels of bacteria and toxins, and improved CSF circulation. Some patients are able to pass this plan to reduce the risk of late reoperation, especially children due to the high plasticity of the CSF circulation system. In this study, 6 cases (5 children) underwent drainage treatment, following the clamping of the EVD tube for approximately one week, the head CT scan revealed the absence of ventricular dilatation and hydrocephalus symptoms, subsequently removed the EVD tube, and no VPS was required (6/35; 17.14%). In addition, adequate clearance of CSF can greatly reduce complications after subsequent shunting. In the present group of 35 cases, 28 cases underwent permanent VPS after mVPS, and the failure rate was 7.14% (2/28), which was lower than the failure rate of 8.8–25.6% reported previously^24–26^.

When mVPS is applied to temporary EVD, it may also increase the convenience of caring for the patient, including: (1) Shunt placement using the subcutaneous sheath fixed on the scalp, to counter the pressure of the drainage tube, the drainage tube is not easy to slip out by accident. (2) The shunt pump regulates pressure to avoid excessive CSF drainage, and regularly pressing the shunt pipe storage sac helps determine whether the tubing is blocked, keeps drainage tube patency, and reduces the risk of diversion pipe blockage. There were only two cases of diversion pipe blockage during drainage in this group. (3) It is convenient for regular retention of CSF specimens for examination, timely detection and prevention of intracranial infection; (4) It avoids repeated lumbar puncture, ventricular drilling and other painful procedures, and reduces the risk of intracranial rebleeding, reducing the medical economic burden, as well as being easily accepted by family members.

**Figure 4:**
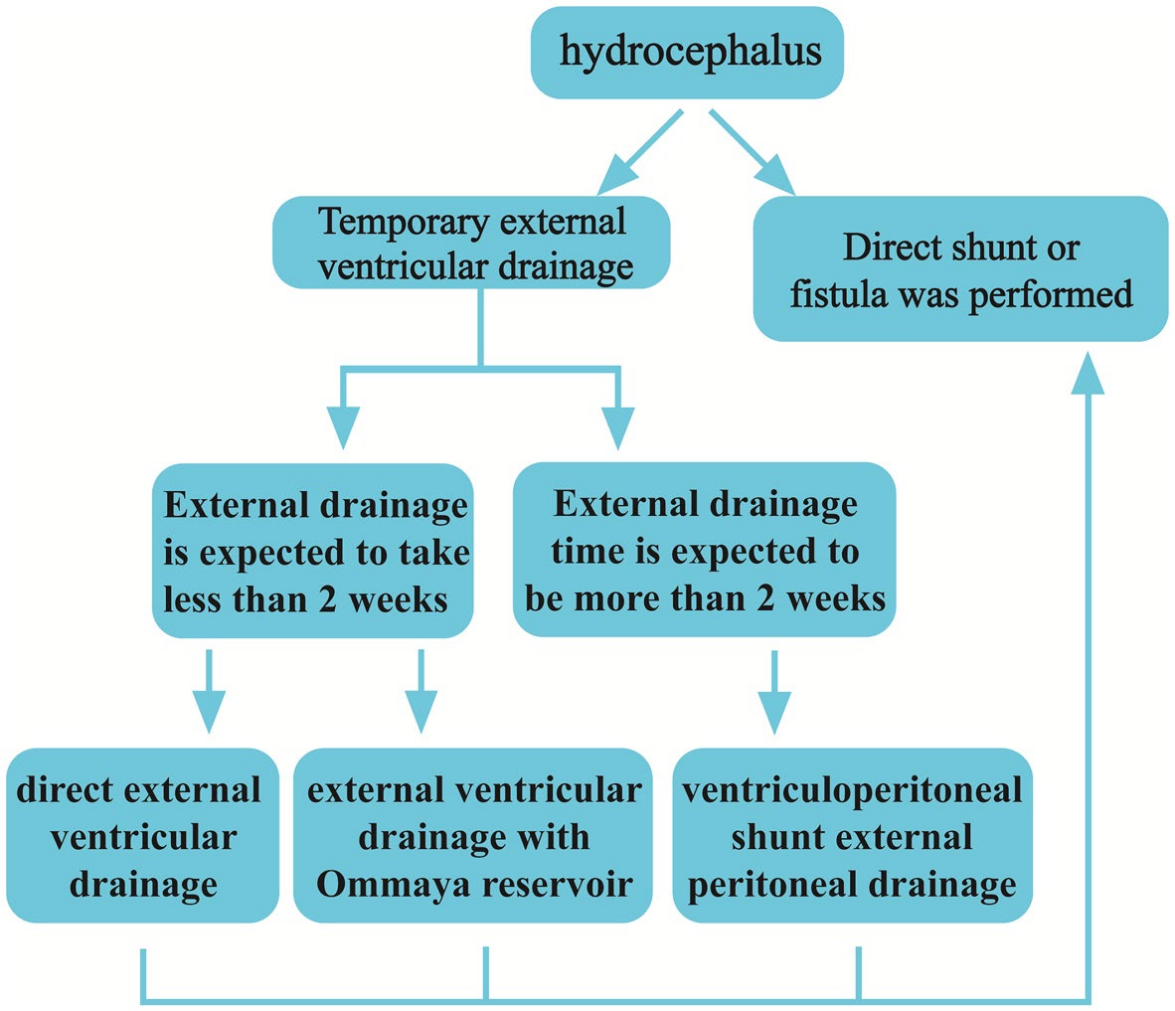
Procedure of surgical selection for patients with temporary external ventricular drainage.

In the present study, patients with hydrocephalus that cannot be solved temporarily by ostomy or other shunt, and who require temporary external drainage to relieve hydrocephalus or improve the nature of CSF, were found to have external drainage time longer than two weeks after the full evaluation of experienced clinical neurosurgeons. Results of this study demonstrate that mVPS is suitable for such patients. Figure 4 shows a flow chart of how to screen such patients, providing a reference for physicians. (Fig. 4) EVD of less than two weeks is generally used for patients with neoplastic or hemorrhagic progressive hydrocephalus who are not amenable to self-improvement or curative surgery, and EVD of more than two weeks is generally used for infective hydrocephalus.

### Limitations

This study is limited to a single-center setting and lacks data pertaining to preterm infants. Our current investigation focuses on the evaluation of treatment strategies at various stages. In future efforts, we aim to carry out a prospective and systematic exploration, extending the follow-up period and evaluating the surgeon's influence on surgical results. To accomplish this, we plan to execute multi-center studies going forward.

## Conclusions

Among surgical procedures for EVD placement, mVPS stands out as a low complication, secure, and reliable method. It effectively addresses the drawbacks of temporary external drainage, such as limited tube placement duration, increased risk of infections, and care complexities, or the necessity for repeated surgeries. It enhances the treatment effect for patients and reduces multiple operation costs, which make mVPS worthy of popularization and routine application for patients who require CSF drainage.

## Data Availability

The datasets used and/or analysed during the current study available from the corresponding author on reasonable request.

## Abbreviations

CSF: Cerebrospinal fluid
dEVD: Direct EVD
EVD: External ventricular drainage
EVDvOR: EVD via Ommaya reservoir
GCS: Glasgow Coma Scale
GOS: Glasgow Outcome Score
LTEVDs: Long-tunneled external ventricular drains
mVPS: Modified ventriculoperitoneal shunt
OR: Ommaya reservoir
VPS: Ventriculoperitoneal shunt
